# *In Vivo* Exposure of Deltamethrin Dysregulates the NFAT Signalling Pathway and Induces Lung Damage

**DOI:** 10.1155/2024/5261994

**Published:** 2024-08-29

**Authors:** Prakriti Sharma, R. S. Sethi

**Affiliations:** Guru Angad Dev Veterinary and Animal Sciences University, Ludhiana, India

## Abstract

Deltamethrin is an insecticide used to control harmful agricultural insects that otherwise damage crops and to control vector-borne diseases. Long-term exposure to deltamethrin results in the inflammation of the lungs. The present study elucidates the molecular mechanism underlying the deltamethrin-induced lung damage. The lung samples were extracted from the Swiss albino mice following the treatment of low (2.5 mg/kg) and high (5 mg/kg) doses of deltamethrin. The mRNA expression of TCR, IL-4, and IL-13 showed upregulation, while the expression of NFAT and FOS was downregulated following a low dose of deltamethrin. Moreover, the expression of TCR was downregulated with the exposure of a high dose of deltamethrin. Furthermore, the immunohistochemistry data confirmed the pattern of protein expression for TCR, FOS, IL-4, and IL-13 following a low dose of deltamethrin exposure. However, no change was seen in the TCR, NFAT, FOS, JUN, IL-4, and IL-13 immunopositive cells of the high-dose treatment group. Also, ELISA results showed increased expression of IL-13 in the BAL fluid of animals exposed to low doses of deltamethrin. Overall, the present study showed that deltamethrin exposure induces lung damage and immune dysregulation via dysregulating the NFAT signalling pathway.

## 1. Introduction

Several pesticides and fertilizers are being used by the farmers to increase productivity as well as to upgrade their economic condition. Pesticides are the chemicals that are mainly used in agriculture or in public health protection programs to protect plants from pests and humans from vector-borne diseases such as malaria, dengue fever, and schistosomiasis [1]. Among different insecticides, pyrethroids are commonly used around the home and in agricultural production to control insects [2]. Pyrethroids are synthetic insecticides derived from natural insecticides pyrethrins of the composite family. These are of two types according to the presence of the alpha-cyano group: type I and type II. Natural pyrethroids are not toxic to mammals; however, synthetically prepared pyrethroids are toxic to mammals [3].

Deltamethrin is frequently used as type II pyrethroid as compared to other pesticides and works even in low concentrations [4]. Pyrethroids change the nerve function of insects by increasing the sodium permeability through acting on voltage-gated sodium channels which results in the destruction of the nervous system [5, 6]. Pyrethroid poisoning results in paralysis and death [7].

Absorption of deltamethrin occurs rapidly through the oral route than the skin [8]. Deltamethrin is readily absorbed from the gastrointestinal tract after oral administration in rats and mice and rapidly metabolized by tissue esterases and liver microsomal oxidases [9]. Studies showed that metabolites of deltamethrin are more toxic than the parent compound deltamethrin. The authors in [10, 11] found that male Wistar rats exposed to deltamethrin for 45 days developed lung inflammation with the increase in macrophages gathering and reactive oxygen species (ROS) concentration.

The nuclear factor of activated T-cells' (NFATs) signalling pathway is responsible for the expression of interferon gamma (IFN-*γ*), interleukin 4 (IL-4), IL-17, and tumor necrosis factor (TNF) in T helper (Th) 1, Th2, and Th17 cells [12] and also controls the morphological maturation of lungs [13]. Pesticide exposure leads to Th2 cell expression related to asthma and allergy [14]. Exposure to deltamethrin causes multiorgan toxicity as well as toxicity to the immune system. Deltamethrin is an immune dysregulator as it has a strong affinity for cluster of differentiation (CD) 4 and CD8 receptors [15]. Deltamethrin exposures decrease splenic T-cell and B-cell populations and suppress cytokines such as IFN-*γ*, IL-2, and IL-4 [16]. Deltamethrin induces brain-derived neurotrophic factor (BDNF) expression by elevating calcium^+2^ (Ca^+2^) influx in neurons and by phosphorylating extracellular signal-regulated kinases that affect neuronal activity in culture and in the rat brain, indicating the possibility of neuronal hyperexcitation if deltamethrin enters the brain [17]. In quail, deltamethrin impacts antioxidant defense which results in cerebrum injury by downregulating nuclear factor erythroid-2-related factor 2 (Nrf2). Deltamethrin exposure also downregulates B-cell lymphoma gene 2 (Bcl-2) level and elevates Jun N-terminal kinase-3, caspase-3, and Bcl-2-associated X expression along with the upregulation of toll-like receptor 4 (TLR4) following inflammation-associated genes inducing inflammation and apoptosis by inhibiting the Nrf2/TLR4 signalling pathway [18]. Moreover, deltamethrin exposure in quails dysregulates Nrf2/TGF-*β*1/SMAD3 pathway and results in liver fibrosis through Nrf2 expression inhibition, inflammation and apoptosis mediated oxidative stress [19].

T-cell receptor (TCR) plays an important role in generating an immune response against disease caused by organisms, cancer cells, and altered self- antigens. It recognizes and binds to the specific protein antigen fragments presented by major histocompatibility complex (MHC) that appeared on the antigen-presenting cells' (APCs) surface further resulting in the intracellular signal activation and triggering an immune response [20]. TCR performs both antigen identification and signal transduction. TCR cohesion is critical for the production of optimal and coordinated immune responses [21].

Pesticide exposures result in the death of immature T-cells [22] and suppress T-cell- and B-cell-facilitated immune response [23] that lead to immune dysregulation [24]. Pesticide exposure reduces cell proliferation and induces apoptosis as well as the activity of cytotoxic T-lymphocytes (CTLs) [25]. A study by the authors in [16] on the deltamethrin-mediated humoral and cell-mediated immunotoxicity showed that exposure to deltamethrin inhibits the expression of cytokines and therefore modifies the functioning of the immune system. A study of differential genes in deltamethrin-exposed zebrafish showed that dysregulation of genes results in cell signalling pathways and nervous system imbalance [26]. Furthermore, an immunotoxin study of deltamethrin in fish showed a decline in lymphocytes and an increase in neutrophil number. Key components (complement (C3), antibodies, and lysozymes) of the immune were decreased, while the enzyme activity of alkaline phosphatase (ALP) was increased along with the dysregulation of TLR signalling genes resulting in suppression of the immune system [27].

Recent studies revealed that NFAT regulates sepsis-induced lung injury via endothelial cell inflammation mediating the development of lung injury [28–30]. Upon T-cell activation, NFAT (transcription factor) present in the cytoplasm gets dephosphorylated and imported to the nucleus where it binds to the promoter resulting in the expression of responsive genes. NFAT plays an important role in the T-cell and B-cell functioning [31]. Out of all 5 members of the NFAT family, NFATc1 plays an important role in the T-lymphocyte development and differentiation of Th2 response and production of its specific cytokines, i.e., IL-4, IL-5, and IL-13. This results in stimulation of IgE production, mucosal mastocytosis, and eosinophils, which are the main cause of lung inflammation [32, 33]. Transcription-factor NFATc1 is important for the differentiation and development of T-cells, cardiac valves, and osteoclasts [34]. Mutation in NFATc1 results in a decrease in T-cells and also a flaw in cardiac growth [35].

cJUN and cFOS are proto-oncogenes [36] form homodimer (cJUN-cJUN and cFOS-cFOS) or heterodimer (cFOS – cJUN) by binding through basic Leucine zipper (bZIP) ([37] and form complex having different DNA binding capacities known as transcriptional factor activator protein 1 (AP-1) [38]. AP-1 cooperates with NFAT for the expression of cytokines and chemokines required for the regulation and functioning of the immune system [39].

IL-4 is an anti as well as anti-inflammatory cytokine [40] that plays a key role in cellular inflammation responsible for the migration of T-lymphocytes, monocytes, basophils, and eosinophils to inflammatory loci by interacting with vascular cell adhesion molecule-1 (VCAM-1) in asthmatic lungs. IL-4 induces an allergic immune response by inhibiting the T-cell apoptosis through the downregulation of Fas expression on the cell which by binding to the Fas ligand induces apoptosis [41]. Pesticide exposure results in various allergic diseases marked by CD4+ T-lymphocytes-mediated type 2 inflammation [42]. Rats exposed to carbyl showed repressed lymphocyte proliferation with the Th1/Th2 imbalance. It decreases the expression of Th1 cytokines (IL-2, IFN-*γ*, IL-1, and TNF-*α*), while the expression of Th2 cytokines (IL-4 and IL-10) is increased which may result in the carbyl-induced allergic, autoimmune, tumor, and infectious disease development [43]. IL-13 is secreted by Th2 cells. The authors in [44, 45] reported that IL-13 overexpression in the lungs induces increased production of mucus, goblet cell hyperplasia, eosinophilic tissue inflammation, fibrosis in the airway, crystal deposition, eotaxin production, atopic diseases, and asthma.

Previous studies show that chronic exposure to various pesticides through the oral route even affects the nontargeted organs such as the lungs and alters the pulmonary transcripts [46–58]. Recently, we reported the dysregulation of the planar cell polarity (PCP) pathway [59], apoptosis pathway [60], Wnt signalling pathway [61], and small lung cancer pathway [62] in mice lung following exposures to fipronil, chlorpyrifos, cypermethrin and 2,4-dichlorophenoxyacetic acid (2, 4-D). Carp exposed to chlorpyrifos results in TCR dysfunctioning with the development of tissue inflammation and oxidative stress [63].

Earlier research has shown that oral exposure to deltamethrin causes lung inflammation and affects the immune system by impairment of proinflammatory cytokines in the Swiss albino mice [64]. TCR plays an important role in the activation of T-cells. For the adaptive immune system against the various encountered antigen there is diversity in the T-cell population [65, 66]. In the current study, the role of TCR, NFAT, FOS, JUN, IL-4, and IL-13 in immune-dysregulation-mediated lung damage was studied by transcriptomic and translational analyses of the lung sections following low and high doses of deltamethrin.

Deltamethrin exposure results in lung damage but the molecular mechanism for deltamethrin-induced lung damage is unknown. Therefore, we hypothesized that deltamethrin induced lung damage by dysregulating the immune system and the NFAT signalling pathway plays an important role in T-cell proliferation [34]. So, we selected the genes (TCR, NFAT, FOS, JUN, IL-4, and IL-13) of the NFAT signalling pathway to study the role of the immune system in the deltamethrin-induced pulmonary damage and we present the maiden data on the NFAT signalling pathway and gene expression in the lungs of mice following exposure to deltamethrin.

## 2. Materials and Methods

### 2.1. Molecular Docking

The docking was performed by using the SwissDock tool [67]. The native ligand for protein structures was deltamethrin whose structure was available in the Zinc database [68]. The proteins selected for the docking were TCR (1TCR) and FOS (2WT7) whose structures were retrieved from the Protein Data Bank (PDB) [69]. Furthermore, the energy minimization of the PDB protein structures was performed by using the online tool ModRefiner [70]. The 2D structure of protein-ligand interaction was analysed using the chimera tool [71]. After docking analysis, further study has been performed to investigate the transcription and quantification of genes of the NFAT signalling pathway in lungs following the exposure to low and high doses of deltamethrin.

### 2.2. Chemicals

Deltamethrin ((s)-*α*-cyano-3-phenoxybenzyl-cis-(lR)-cis-3-(2,2- dibromovinyl)-2,2-dimethylcyclopropanecarboxylate) PESTANAL® (catalogue no. 45423) purity 98.6% and corn oil (catalogue no. C8267) were obtained from Sigma Aldrich, Bangalore. cDNA kit (catalogue no. 205313) and the reverse transcription kit were the QuantiTect®Reverse Transcription kit. Antibody: primary antibodies were raised in rabbits viz. TCR *β* (catalogue no. 5651-30T) from BioVision. NFAT2 (catalogue no. ITT0446), cFOS (catalogue no. ITT00132), cJUN (catalogue no. ITT06090), IL-4 (catalogue no. ITT05142), and IL-13 (catalogue no. ITT06813) were purchased from Immunotag, and secondary antibody (anti-rabbit) from SIGMA. The DAB substrate kit (catalogue no. SK4100) was from the Vector Lab.

### 2.3. Experimental Animals

The experiments were carried out at Guru Angad Dev Veterinary and Animal Sciences University, Ludhiana as per guidelines of the Committee for the Purpose of Control and Supervision of Experiments on Animals (CPCSEA) with reference number: GADVASU/2020/IAEC/56/06. Swiss albino healthy male mice (*n* = 18) aged 6–8 weeks were purchased from the Paradise Rabbit Farm, Kurukshetra, Haryana. Before treatment, mice were kept for a week in the experimental facility for adaptation. Mice were kept under controlled conditions with 12 h light and dark cycles in polypropylene cages at institutional small animal houses. Mice were fed synthetic pelleted mice feed obtained from Rodent Research India Pvt. Ltd., Jind, Haryana.

### 2.4. Experimental Design

Animals were weighed before treatment and then randomly divided into three groups, one control group served as negative control and received solvent (corn oil) and two treatment groups received 1/20^th^ LD_50_, i.e., 2.5 mg/kg body weight (low dose) dose and 1/10^th^ LD_50_, i.e., 5 mg/kg body weight (high dose) of deltamethrin dissolved in corn oil orally for 90 days. LD_50_ of deltamethrin is 50 mg/kg body weight [72]. Immediately on 91 days of trial, each group was anaesthetized with 1/10^th^ of the actual dose of xylazine and ketamine combination [62] and sacrificed for sample collection.

### 2.5. Body Weight

The body weights of all the mice groups were measured on 0^th^ day, 45^th^ day, and 90^th^ day.

### 2.6. Sample Collection

Blood was collected by cardiac puncture for serum isolation and bronchioalveolar lavage (BAL) fluid was collected from the left lung. Both serum and BAL fluid samples were stored at −80°C for further analysis. The right lung is chopped into small sections and kept at −80°C in RNA solution till further use. The left lung was fixed in the 4% paraformaldehyde and stored at 4°C for 12 hours prior to immunohistochemical analysis.

### 2.7. Quantitative Real-Time PCR

The total RNA was extracted from the lung samples by using the TRIzol method (Ambion, Life Technologies, USA). After that, the quality and quantity of RNA were checked using an ultraviolet light nanodrop spectrophotometer (Thermo, USA), which was further used for a cDNA synthesis using a cDNA synthesis kit (QIAGEN) according to the given protocol in the kit. The concentration of RNA used for cDNA synthesis was 1000 ng/*μ*l. For real-time PCR, already published primers were used (Table 1). Then, the fold change was calculated using the ∆∆CT method [80].

### 2.8. Immunohistochemistry

Immunohistochemistry was performed to check the protein expression of TCR*β*, NFATc1, FOS, JUN, IL-4, and IL-13 in the lung. Paraffin blocks were prepared to get 5 *μ*m thick sections. The protocol for immunohistochemistry was described previously [81]. In brief, the procedure is as follows. The tissue sections were deparaffinized in xylene followed with the rehydration by using gradient ethanol. Tissue sections were incubated with 3% H_2_O_2_ in the dark chamber to inhibit the cell's own peroxidase and proceeded with the boiling in Tris EDTA and 1X PBS for antigen retrieval. Then, the slides were incubated with 1% BSA in the dark chamber for 1 hour followed by incubation of slides at 4°C with primary anti-TCR, NFATc1, cFOS, JUN, IL-4, and IL-13 antibodies raised in rabbit for overnight. On the next day, after washing with 1X PBS, slides were incubated with secondary HRP-tagged anti-rabbit antibody for half an hour in the dark chamber. The immunopositive reaction was observed with the color development step using chromogen (Vector laboratories, cat no. SK-4100) followed by counterstaining with hematoxylin which specifically stained the nuclei of the cell. IHC control was stained without primary or secondary antibodies or both.

### 2.9. Immunopositive Scoring for Immunohistochemistry

Immunohistochemical-stained slides were used for the quantification of TCR, NFAT, JUN, FOS, IL-4, and IL-13 immunopositive cells. To ensure the regularity mentioned previously, the cells were physically counted using the microscope's 40x objective lens in 10 fields/slide. Immunopositive cells from six animals per group were calculated. Furthermore, the cells were compared between the control and treatment groups [59].

### 2.10. ELISA

The comparison for the presence of AP-1, IL-4, and IL-13 in the serum and BAL fluid samples after the long-term exposure of deltamethrin by using sandwich Enzyme Linked Immunosorbent Assay (ELISA) was performed according to the manufacturer's protocol. Furthermore, the absorbance of the samples was recorded and used to calculate the concentration of the target protein and further compared between the control and treatment samples.

### 2.11. Statistical Analysis

One-way ANOVA (one-way analysis of variance) was performed to find the statistical difference (*p* < 0.05) for the immunopositive scoring and fold change (mRNA expression) between the groups following exposure to low and high doses of deltamethrin by using GraphPad Prism 8 software.

## 3. Results

### 3.1. Protein and Ligand Interaction

The primary molecular docking analysis was performed using the SwissDock online server and further analysis was performed with the Discovery Studio tool [82] to identify the interactions between the target proteins and the deltamethrin ligand.

**Figure 1:**
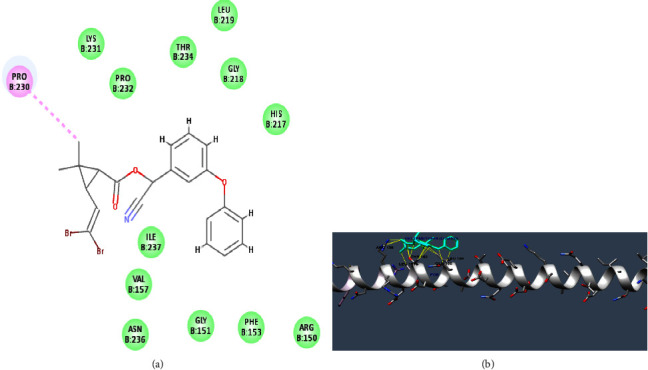
Molecular docking analysis to identify the interactions between the target proteins and the deltamethrin ligand: (a) molecular docking showing molecular interactions between deltamethrin and TCR and (b) molecular docking analysis showing the interaction between cFOS and deltamethrin using SwissDock tool.

Van der Waals contacts were seen at the following residues for the TCR (T-cell receptor) following molecular docking with deltamethrin: PRO 231, LYS 231, THR 234, LEU 219, GLY 21, HIS 217, ILE 237, VAL 157, ASN 236, GLY 151, PHE 153, and ARG 150. Furthermore, at the PRO 230 residue, an alkyl interaction was observed (Figure 1(a)). 37 clusters in total were found by docking analysis with binding free energy (ΔG) of −8.76253 kcal/mol at the first cluster rank (Table 2).

ARG 158, THR 162, LEU 161, GLN 166, and LEU 165 amino acid positions were the sites of molecular interactions revealed by the SwissDock analysis of the cFOS protein and deltamethrin ligand with an estimated binding free energy (ΔG) of −6.6688457 kcal/mol (Figure 1(b)). A total of 31 clusters were found by the docking analysis for deltamethrin and cFOS (Table 2).

The structural analysis provided evidence for the presence of molecular interactions between the target protein molecules and respective deltamethrin ligand molecules. The significance of the interacting amino acid residues in the molecular recognition process is shown by the way in which these interactions together influence deltamethrin's binding affinity and specificity for the TCR and cFOS proteins which provide light on functional implications of the deltamethrin binding with these protein molecules.

### 3.2. Body Weight

**Figure 2:**
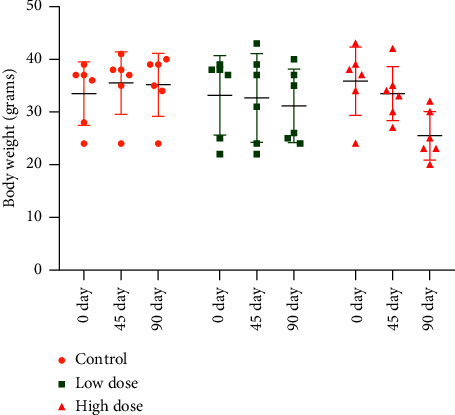
Body weight (grams) of mice following exposure to low (2.5 mg/kg) and high doses (5 mg/kg) of deltamethrin (*n* = 6) (mean ± SD). SD: standard deviation.

The body weight of the animal was decreased following the high dose of deltamethrin, while there was no change in the weight of the control group animal and animals treated with a low dose of deltamethrin (Figure 2).

### 3.3. mRNA Expression of Genes of the NFAT Signalling Pathway

**Figure 3:**
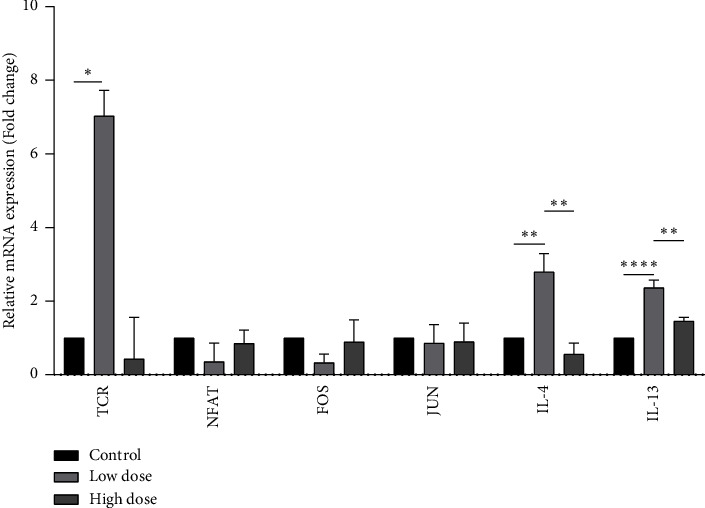
Relative mRNA expression of TCR, NFAT, FOS, JUN, IL-4, and IL-13 following the exposure to low (2.5 mg/kg) and high doses (5 mg/kg) of deltamethrin. ^∗^*p* < 0.05 (significant), ^∗∗^*p* < 0.01 (moderately significant), and ^∗∗∗∗^*p* < 0.0001 (highly significant). *p* value: probability value.

The pulmonary mRNA expression of TCR, IL-4, and IL-13 was significantly increased by 2.8 (*p* < 0.05), 1.48, and 1.25 folds (*p* < 0.05) following a low dose of deltamethrin as compared to the control. However, high-dose treatment decreases the expression of TCR by −1.2. The expression of NFAT and FOS was also downregulated by −1.49 and −1.6 folds following a low dose of deltamethrin (Figure 3).

### 3.4. Protein Expression of the Genes of the NFAT Signalling Pathway

**Figure 4:**
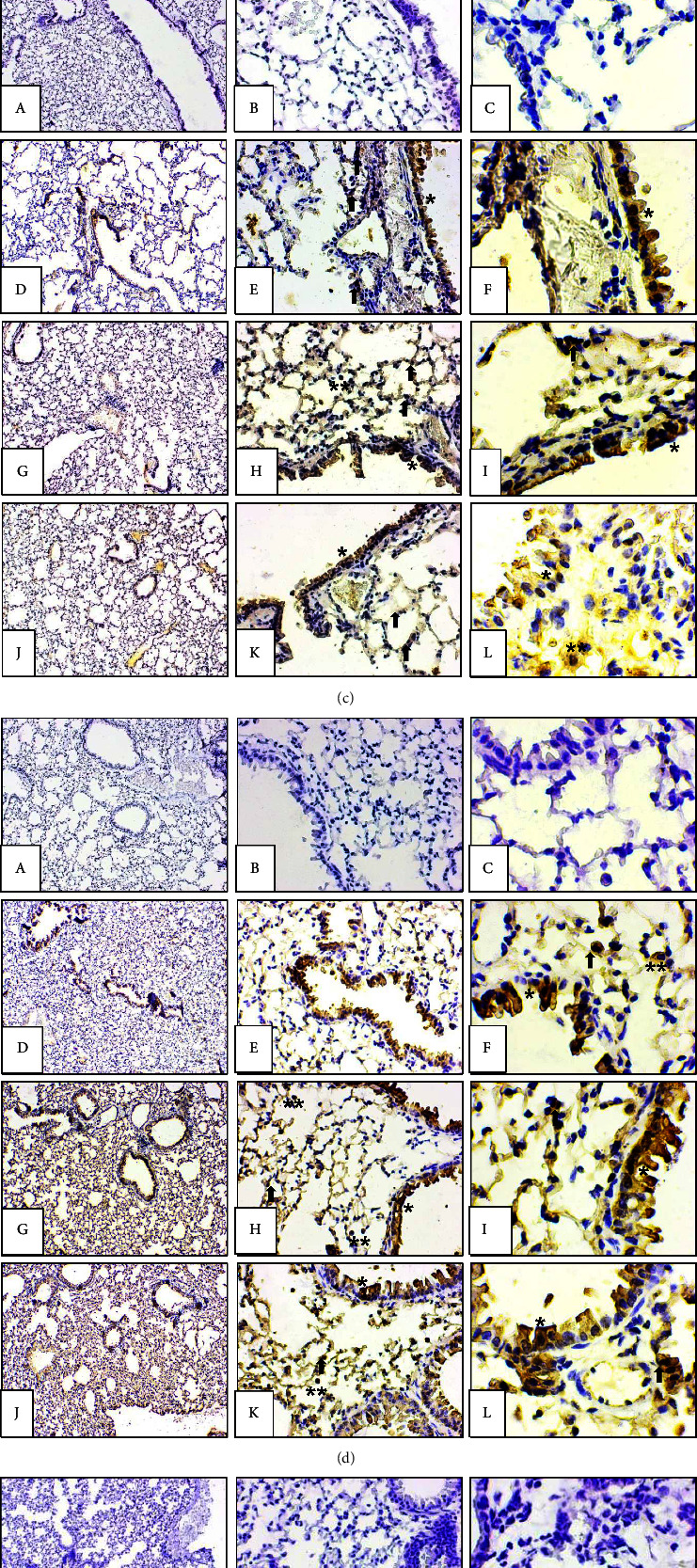
Lung sections stained with (a) TCR, (b) NFAT, (c) FOS, (d) JUN, (e) IL-4, and (f) IL-13 primary antibodies. Sections without primary antibodies (A, B, C) resulted in no color development. Immunopositive cells in airway epithelium (pentagon), alveolar epithelium (star), alveolar septal cells (arrow), and macrophages (double) in the control group (D, E, F) and low-dose (2.5 mg/kg) (G, H, I) and high-dose (5 mg/kg) (J, K, L) groups. Original magnification A, D, G, J: 10x; B, E, H, K: 40x; C, F, I, L: 100x.

**Figure 5:**
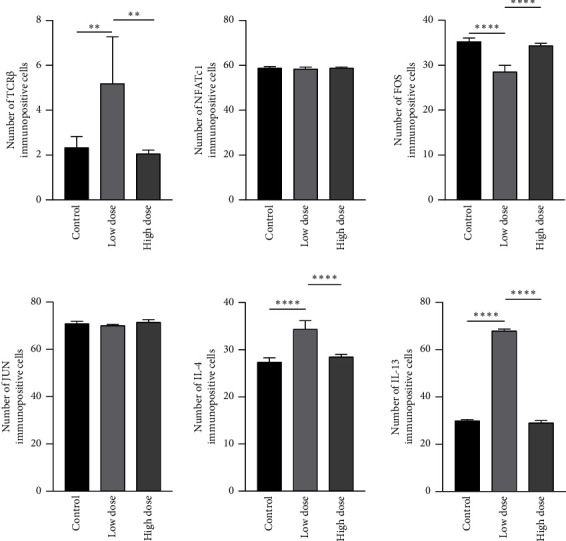
Immunopositive scoring of TCR, NFAT, FOS, JUN, IL-4, and IL-13 (mean ± SD) for immunohistochemical-stained lung tissue sections in control and treatment groups. ^∗∗^*p* < 0.01 (moderately significant) and ^∗∗∗∗^*p* < 0.0001 (highly significant). *p* value: probability value; SD: standard deviation.

The protein expression of TCR, NFAT, FOS, JUN, IL-4, and IL-13 was analysed and quantified by immunohistochemistry. A low dose of deltamethrin exposure showed strong TCR immunopositive reaction in the airway epithelial (Figure 4(a), G, H, I) and IL-4 (Figure 4(e), G-I) and IL-13 (Figure 4(f), G, H, I) immunopositive reaction in the alveolar epithelium, septal cells and macrophages along with the significant increase in the number of TCR, IL-4 and IL-13 immunopositive cells as compared to the control group (Figure 5). Furthermore, the NFAT (Figure 4(b), G, H, I) and FOS (Figure 4(c) G, H, I) immunopositive cells also showed a strong immunopositive reaction in alveolar epithelial cells, septal cells, and macrophages but the immunopositive scoring of the FOS and NFAT immunopositive cells showed significant decreases only in the FOS immunopositive cells as compared to the control following a low dose of deltamethrin. Moreover, low dose as well as high dose of deltamethrin exposure showed strong JUN immunopositive reaction in the airway epithelial, septal cells, and macrophages (Figure 4(d), D, E, F, G, H, I, J, K, L). But there was no change in the JUN immunopositive cells in both dosage groups (Figure 5). However, the high-dose treatment group showed no change in the TCR, NFAT, FOS, JUN, IL-4, and IL-13 immunopositive cells (Figure 5).

### 3.5. ELISA

**Figure 6:**
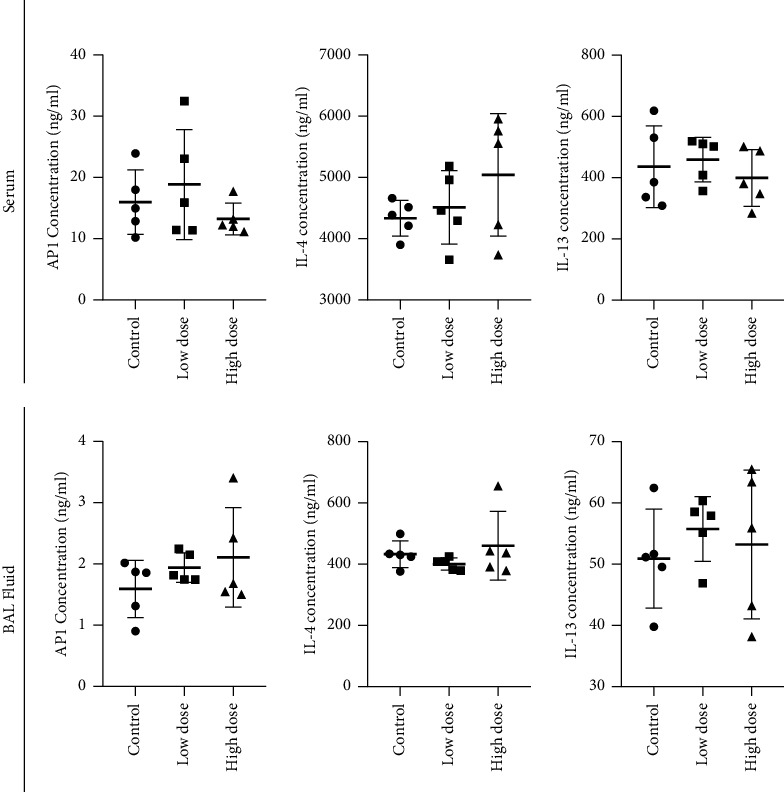
AP-1, IL-4, and IL-13 concentrations in serum and bronchial lavage fluid (BAL) (mean ± SD) of mice following exposure to low (2.5 mg/kg) and high doses (5 mg/kg) of deltamethrin. SD: standard deviation.

The ELISA was employed to contrast the AP-1, IL-4, and IL-13 concentrations in the serum and BAL fluid of the control and treated mice. There was no difference in the concentration of AP-1 and IL-4 in both sera as well as in the BAL fluid samples between the control and deltamethrin-treated mice (Figure 6). However, an increase in the concentration of IL-13 was observed in the BAL fluid samples of the low-dose deltamethrin-treated mice (Figure 6) but no change in the concentration of IL-13 was observed in the serum following low- and high-dose treatment of deltamethrin (Figure 6).

## 4. Discussion

Deltamethrin exposure stimulates the formation of extracellular traps by elevating the levels of myeloperoxidase (MPO) and reactive oxygen species (ROS) along with mitochondrial dysfunctioning leading to immunotoxicity in Manila clam (*Ruditapes philippinarum*) hemocytes [83]. Acute exposure to deltamethrin induces ROS production which results in DNA damage, immune and neurotoxicity in the DM induces DNA damage, immunotoxicity, and neurotoxicity into brown trout brain tissue [84]. Deltamethrin treatment in male albino rats decreases various blood cells and antioxidant enzymes in different tissues of rats along with uric acid levels and increases transaminase and lactate dehydrogenase enzyme activity in serum, along with the extent of liver, kidney, and brain thiobarbituric acid reactive substances. Also, the level of systemic inflammatory and systemic immune-inflammatory markers was increased and the histological lesion was observed in the liver, kidney, and brain drawing attention to the possible negative effects of deltamethrin [85, 86] found that deltamethrin induces cardiotoxicity and hepatotoxicity by a complex mechanism. It was initially discovered that the NFAT regulated the transcription of activated T-cells [87].

NFAT signalling pathway is regulated by the intracellular concentration of Ca^2+^ coupled with TCR activation [88]. Our results showed that a low dose of deltamethrin upregulated the expression of TCR, while a high dose of deltamethrin decreases the mRNA expression of TCR (Figure 1). A high number of TCRs results in the activation of T-cells and makes T-cells more sensitive toward the antigen. T-cells with less number of TCRs fail to activate because less number of TCRs means insufficient signals for activation of the antigen, but T-cells expressing a high number of TCRs result in the activation of T-cells and make T-cells more sensitive toward the antigen [89]. A constitutive TCR expression decreases TCR efficiency [43] and decreased TCR efficiency favored Th2 differentiation and IL4 expression [90]. TCR is required for T-cell activation but a downregulation in the components of the TCR complex inhibits T-cell activation [91]. TCR downregulation may be for the protection of cells from excessive T-cell activation [92]. Downregulation and defects in TCR signalling are associated with dysfunctioning and hyporesponsiveness in T-cells suggesting immune modulations [93].

NFAT plays an important role in the differentiation and development of T-cells [34, 94] and induces the expression of cytokines such as IL-4, IL-5, and IL-13 upon TCR stimulation [95]. The NFAT transcription factor is an oncogene [96]. A low dose of deltamethrin downregulated the NFAT as well as FOS expression (Figure 1) which may suggest that there may be alternative NFAT-activation pathways mediated by some other pathways such as IL-7 as IL-7-dependent activation of JAK3 led to tyrosine phosphorylation and activation of NFATc1 [97]. Adrenal corticosteroids are used to decrease the expression of cFOS during asthma which is desired to overcome lung inflammation [98]. In the present study, a decrease in the mRNA expression of FOS suggests that there will be a limited formation of the AP-1 complex which will dysregulate the NFAT signalling pathway. A decrease in the JUN/AP-1 expression alters the target cytokines (IL-4, IL-5, and GM-CSF) expression by the posttranscriptional and translational modifications [99]. Taken together, data suggest dysregulation of the NFAT signalling pathway following exposure to a low dose of deltamethrin. Also, a decrease in the expression of NFATc1 and FOS might be a compensatory mechanism for the cytokine expression and inflammatory response during low doses of deltamethrin-induced lung damage.

IL-4 acts as an anti-inflammatory as well as proinflammatory cytokine [40] which plays an important role in the production of Th2-specific cytokines (IL-4, IL-5, and IL-13) by the differentiation of CD4^+^ T-cells/Th cells to Th2 cells [100]. In the current study, IL-4 expression was upregulated following exposure to a low dose of deltamethrin (Figures 1 and 4(e)). IL-4 upregulation induces lung inflammation characterized by BAL fluid eosinophilia, airway hyperresponsiveness (AHR), and an increase in the number of goblet cells in mouse lungs [101]. The data suggest that IL-4 upregulation might be a protective mechanism during deltamethrin-induced damage.

A study with environmental pesticides on pregnant women showed that upregulation of IL-13 is associated with tissue repair [102]. Moreover, the expression of IL-13 was also upregulated following low dose of deltamethrin (Figures 1 and 4(f)) that might result in the normalization of damage lung due to enhanced activity of tissue repair enzyme, while the high dose did not alter the expression of IL-13 which may suggest the suppression of tissue repair process following exposure to high dose. IL-13 is a hallmark of the pulmonary allergic diseases and is a crucial factor for the allergen-mediated Th2 immune response that results in the various proinflammatory conditions [103]. Overexpression of IL-13 in the lungs elevates the production of mucus, goblet cell hyperplasia, eosinophilic tissue inflammation, fibrosis in the airway, crystal deposition, eotaxin production, atopic diseases, and asthma [44, 45]. The abovementioned study suggested that a low dose of deltamethrin dysregulates the expression of genes to overcome and cure the deltamethrin-induced lung damage but a high dose shuts down the pathway and there was no responsive or repair mechanism to overcome the lung inflammation. Also, it was seen with the animal's body weight which showed that with the high dose, the body weight of the animal decreased. However, with the treatment of low doses, there was no change in body weight.

## 5. Conclusion

The abovementioned study showed that chronic exposure to a low (2.5 mg/kg) dose of deltamethrin altered TCR, NFATc1, FOS, IL-4, and IL-13 expression results in the dysregulation of the NFAT signalling pathway. Along with this, an increase in the IL-13 concentration was observed in the low-dose-treated mice BAL fluid samples. However, a high dose (5 mg/kg) of deltamethrin did not alter the expression of genes. The mice' body weight decreases with high-dose treatment, which may suggest that at the high dose of deltamethrin, repair mechanism of the animal did not work to cure damage resulting in lung inflammation. The immune dysregulation at low-dose and high-dose treatment might be the reason behind lung inflammation. Furthermore, this can be studied in the targeted organs such as the liver and kidney. Also, a knockout study can be performed to study the correlation between the NFAT signalling pathway and lung damage.

## Figures and Tables

**Table 1:** Primer sequences used in real-time PCR analysis.

Name	Primer sequence	Reference
TCR*β*	F: GAGACGGCTGTTTTCCAGAC R: GGCCCAGAGTTTGCTTACAA	[73]

NFATc1	F: GTGGCAGCCATCAACGCCCT R: TACGAGGCCTGTGGCACCGA	[74]

cFOS	F: ACCATGATGTTCTCGGGTTTCAA R: GCTGGTGGAGATGGCTGTCAC	[75]

JUN	F: ACTCGGACCTTCTCACGTCG R: TAGACCGGAGGCTCACTGTG	[76]

IL-4	F: CCAGCTAGTTGTCATCCTGCTCTTCTTTCTCG R: CAGTGATGTGGACTTGGACTCATTCATGGTGC	[77]

IL-13	F: TGAGCAACATCACACAAGACC R: AGGCCATGCAATATCCTCTG	[78]

*β*-actin (mouse)	F: CTGTCCCTGTATGCCTCTG R: ATGTCACGCACGATTTCC	[79]

**Table 2:** Binding energy and full-fitness value of the docked complex.

	Number of SwissDock cluster	Full fitness (kcal mol) (−)	Estimated *δ*G (kcal mol) (−)	Cluster rank	
TCR	37	1859.4634	8.76253	1	0
		1858.1428	8.670424	1	1
		1854.614	8.506084	1	2
		1850.9839	8.171686	1	3
		1849.8317	8.743343	1	4
		1849.752	8.736348	1	5
		1849.5947	8.714316	1	6
		1848.4362	8.760416	1	7

cFOS	31	756.971	6.6688457	0	0
		756.971	6.6688457	0	1
		756.956	6.668018	0	2
		756.956	6.668018	0	3
		756.8105	6.6408553	0	4
		756.8105	6.6408553	0	5
		756.8105	6.6408553	0	6
		756.8105	6.6408553	0	7

## Data Availability

The data used to support the findings of this study are included within the article. There is no additional source of data.
