# Correction to “*In Vivo* Exposure of Deltamethrin Dysregulates the NFAT Signalling Pathway and Induces Lung Damage”

**DOI:** 10.1155/jt/9796826

**Published:** 2025-09-30

P. Sharma and R. S. Sethi, “*In Vivo* Exposure of Deltamethrin Dysregulates the NFAT Signalling Pathway and Induces Lung Damage,” *Journal of Toxicology* 2024 (2024): 5261994, https://doi.org/10.1155/2024/5261994.

In the article, there are errors in the Results and Discussion. In the Results section,

“The pulmonary mRNA expression of TCR, IL-4, and IL-13 was significantly increased by 2.8 (*p* < 0.05), 1.48, and 1.25 folds (*p* < 0.05) following a low dose of deltamethrin as compared to the control. However, high-dose treatment decreases the expression of TCR by −1.2. The expression of NFAT and FOS was also downregulated by −1.49 and −1.6 folds following a low dose of deltamethrin (Figure 3).”

It should read as

“The pulmonary mRNA expression of TCR, IL-4, and IL-13 was significantly increased by 7.03 (*p* < 0.05), 2.79, and 2.37 folds (*p* < 0.05) following a low dose of deltamethrin as compared to the control. However, high-dose treatment decreases the expression of TCR by 0.43 folds. The expression of NFAT and FOS was also downregulated by 0.36 and 0.33 folds following a low dose of deltamethrin (Figure 3).”

In the Discussion section:

“Acute exposure to deltamethrin induces ROS production which results in DNA damage, immune and neurotoxicity in the DM induces DNA damage, immunotoxicity, and neurotoxicity into brown trout brain tissue [84].”

It should read as

“Acute exposure to deltamethrin induces ROS production which results in DNA damage, immune and neurotoxicity in the deltamethrin induces DNA damage, immunotoxicity, and neurotoxicity into brown trout brain tissue [84].”

“Our results showed that a low dose of deltamethrin upregulated the expression of TCR, while a high dose of deltamethrin decreases the mRNA expression of TCR (Figure 1).”

It should read as

“Our results showed that a low dose of deltamethrin upregulated the expression of TCR, while a high dose of deltamethrin decreases the mRNA expression of TCR (Figure 3).”

“A low dose of deltamethrin downregulated the NFAT as well as FOS expression (Figure 1) which may suggest that there may be alternative NFAT-activation pathways mediated by some other pathways such as IL-7 as IL-7-dependent activation of JAK3 led to tyrosine phosphorylation and activation of NFATc1 [97].”

It should read as

“A low dose of deltamethrin downregulated the NFAT as well as FOS expression (Figure 3) which may suggest that there may be alternative NFAT-activation pathways mediated by some other pathways such as IL-7 as IL-7-dependent activation of JAK3 led to tyrosine phosphorylation and activation of NFATc1 [97].”

“In the current study, IL-4 expression was upregulated following exposure to a low dose of deltamethrin (Figures 1 and 4(e)).”

It should read as

“In the current study, IL-4 expression was upregulated following exposure to a low dose of deltamethrin (Figures 3 and 4(e)).”

“The data suggest that IL-4 upregulation might be a protective mechanism during deltamethrin-induced damage.”

It should read as

“The data indicate that exposure to low dose of deltamethrin dysregulates the IL-4 expression, which may further aggravate the lung inflammation.”

“Moreover, the expression of IL-13 was also upregulated following low dose of deltamethrin (Figures 1 and 4(f)) that might result in the normalization of damage lung due to enhanced activity of tissue repair enzyme, while the high dose did not alter the expression of IL-13 which may suggest the suppression of tissue repair process following exposure to high dose.”

It should read as

“Moreover, the expression of IL-13 was also upregulated following low dose of deltamethrin (Figures 3 and 4(f)) that might result in the normalization of damage lung due to enhanced activity of tissue repair enzyme, while the high dose did not alter the expression of IL-13 which may suggest the suppression of tissue repair process following exposure to high dose.”

We apologize for these errors.

